# Appropriateness of empiric antimicrobial therapy with imipenem/colistin in severe septic patients: observational cohort study

**DOI:** 10.1186/s12941-018-0292-7

**Published:** 2018-11-16

**Authors:** Ahlem Trifi, Sami Abdellatif, Cyrine Abdennebi, Foued Daly, Rochdi Nasri, Yosr Touil, Salah Ben Lakhal

**Affiliations:** 1 Medical Intensive Care Unit, University Hospital Center La Rabta, La Rabta Jebbari, 1007 Tunis, Tunisia; 2 Faculty of Medicine, University Tunis, El Manar, Tunis, Tunisia

**Keywords:** Empiric, Antimicrobial, Nosocomial sepsis, Imipenem, Colistin, Intensive care, Outcome

## Abstract

**Background:**

Empiric antimicrobial therapy (EAMT) using imipenem/colistin is commonly prescribed as a first line therapy in critically ill patients with severe sepsis. We aimed to assess the appropriateness of prescribing imipenem/colistin as EAMT in ICU patients.

**Methods:**

A 3-year observational prospective study included ICU patients that required imipenem/colistin as EAMT. The EAMT was assessed according to microbiological and clinical outcomes. The outcomes were: delay in apyrexia, delay in the decrease of the biological inflammatory parameters (BIP), the requirement for vasoactive agents, bacteriological eradication, length of stay, ventilator days and 30-day mortality.

**Results:**

79 administrations of EAMT in 70 patients were studied. EAMT was appropriate in 52% of the studied cases. An ICU stay > 6 days was related to inappropriateness, and chronic respiratory failure was associated with appropriateness. In the appropriate EAMT group, we showed: earlier apyrexia, shorter delay in the decrease of the BIP and a reduced significant vasopressors requirement. Furthermore, EAMT improved survival with a median gain of 4 days. Inappropriate EAMT increased the mortality risk by six. The acquisition of NI in ICU was also an independent factor of mortality.

**Conclusions:**

EAMT using imipenem-colistin was appropriate in half of the cases and inappropriateness was associated with an increased ICU mortality risk.

## Background

Intensive care unit (ICU) acquired nosocomial infections (NI) have increased rapidly in recent years [1]. EPIC II study in 2007 [2] reported a prevalence of 51% significantly higher than the previous EPIC I study of 20% [3]. The pathogens which cause the most are multi-drug-resistant (MDR) Gram-negative bacilli (GNB) [4]. Despite the extreme variation of antimicrobial resistance rates between hospitals and countries, there is significant evidence that resistance rates are steadily increasing [5–7]. Worldwide as in Tunisia, three ‘’leaders’’ of MDR-GNB were often reported: *Enterobacteria* (mainly the extended-spectrum beta lactamase (ESBL) secreting and emergence of carbapenemase-producing strains)*, Acinetobacter* spp. and *Pseudomonas aeruginosa* [4, 8–13].

Infections due to these microorganisms are associated with a prolonged ICU stay, longer ventilator days, higher mortality and increased health care costs [1, 4] with very limited therapeutic options [9, 12–14]. In ICU patients, the occurrence of nosocomial sepsis without bacterial documentation should indicate an early Empiric Antimicrobial Therapy (EAMT). The choice of EAMT is based on the local ecology and the susceptibility profile of isolates. Therefore, EAMT must target the most frequent MDR-GNB previously cited. At present, therapeutic options include carbapenems and polymyxins. Carbapenems are a heterogeneous group of β-lactams with a very broad anti-bacterial spectrum and constitute an unavoidable part of EAMT in severe NI. Their bactericidal activity is related to the connection to penicillin binding proteins, resulting in lysis of endotoxins secreted by GNB [15]. Polymyxins are considered as “old generation” but their therapeutic use has undergone renewed interest owing to the emergence of MDR strains. Its mechanism of action, not fully elucidated, is linked to a polycationic effect disorganizing the phosphate groups of lipopolysaccharides of the GNB’s membrane. The association between imipenem provides a synergy of action and thus improves the bactericidal effect. But what about the clinical impact of this association, the most common prescribed EAMT in ICU patients with severe NI?

Herein, we aimed to analyze the characteristics of EAMT with imipenem-colistin and to evaluate its impact on clinical and microbiological outcomes.

## Patients and methods

### Study design

A prospective observational cohort study with analytical approach over a 36-month period (June 2014 to June 2017). The study was in accordance with the ethical standards of the declaration of Helsinki and approved by the local ethics committee of la Rabta hospital. Given the non-interventional nature, informed consent was not required. All patients or their families were informed of their participation in the study.

### Studied population

Studied population were enrolled, all patients older than 18 years hospitalized in our ICU for at least 48 h and required EAMT with imipenem/colistin. The criteria for suspicion of NI caused by MDR-GNB were: (1) severe sepsis in a patient without known infection, (2) severe sepsis in a patient treated by antimicrobials other than imipenem/colistin, (3) persistence of a fever (> 38 °C) or hypothermia (< 36 °C) with an increase of biological inflammatory parameters in a patient receiving antimicrobials well conducted for at least 72 h.

Microbiological samples were taken before the administration of imipenem/colistin. Subsequently, the patients were analyzed in two groups according to whether this EAMT with imipenem/colistin was appropriate versus not. Patients who died within 72 h of inclusion were excluded.

### Definitions

The different septic states (severe, severe sepsis and septic shock) were defined according to 2014s guidelines [16] given that the study was started before the updating of sepsis-2016.

EAMT was considered appropriate if it targeted, effectively, an MDR-GNB susceptible to imipenem/colistin accompanied with an improvement of infectious signs. In the absence of bacteriological documentation, improvement of infectious signs defined the appropriateness nature. As a result, this EAMT was kept. EAMT was considered inappropriate in the following situations:—the isolated microorganism as the cause of NI was resistant to imipenem/colistin,—worsening of infectious signs due to other agents (non-GNB or yeasts),—worsening of infectious signs without microbiological documentation,—the isolated microorganism had a multi-sensitive profile leading to de-escalation. Therefore, EAMT was changed, extended or de-escalated. Improvement of the clinical signs was judged on apyrexia and the regression of the infectious signs within 72 h of initiating EAMT.

### Microbiological techniques

All isolates from various samples (blood cultures, urine, protected distal samples, sputum, drains and catheters…) were included. The strains were isolated on blood agar, chocolate agar, cemetol agar, King A and King B agar. The identification was performed according to standard microbiology techniques (Gram stain, oxidase and mobility) and biochemical characters using API 20NE galleries (BioMérieux, France). Sensitivity to imipenem was determined by the standard Mueller–Hinton agar diffusion method according to the CA-SFM recommendations. The antibiograms were read using the automated Osiris system. Intermediate susceptibility isolates to imipenem were classified as resistant.

Sensitivity to colistin was determined by the E test, and the isolated strain was considered sensitive when the minimum inhibitory concentration (MIC) was less than 2 mg/L. Microbiological eradication was defined as a negative culture.

### Data collection

We recorded and followed all bacteriological and biological data for the patients as well as the issue of EAMT: maintained, changed, extended or de-escalated and the reasons for modification and patient’s outcome.

### Outcome’s criteria

Outcome’s Criteria were delay in apyrexia, decrease of inflammatory parameters, vasoactive agent requirements, bacteriological eradication, length of stay, ventilator days and 30-day mortality.

### Statistical analyzes

Quantitative variables were expressed as a mean and standard deviation (SD) or a mean and interquartile range [IQR 25th–75th] and compared using the Student’s t-test or ANOVA (analysis of variance) method. Categorical variables were expressed as percentages and compared using the Χ^2^ test or Fisher’s exact test as appropriate.

Logistic regression was used to evaluate the factors associated with appropriate EAMT and between appropriate EAMT and survival. The covariates included were: age, sex, severity scores, medical versus non-medical admission, co-morbidities, shock, mechanical ventilation, and other parameters. The results are expressed in odds ratio (OR) with a 95% confidence interval (CI). The survival analysis was processed by the Kaplan–Meier curves and compared by the Log-Rank test. A p value < 0.05 was fixed for significance. Data analysis was performed using the Statistical Package for Social Sciences (SPSS) version 2.0 software.

## Results

### Study flowchart and patient’s characteristics

**Fig. 1:**
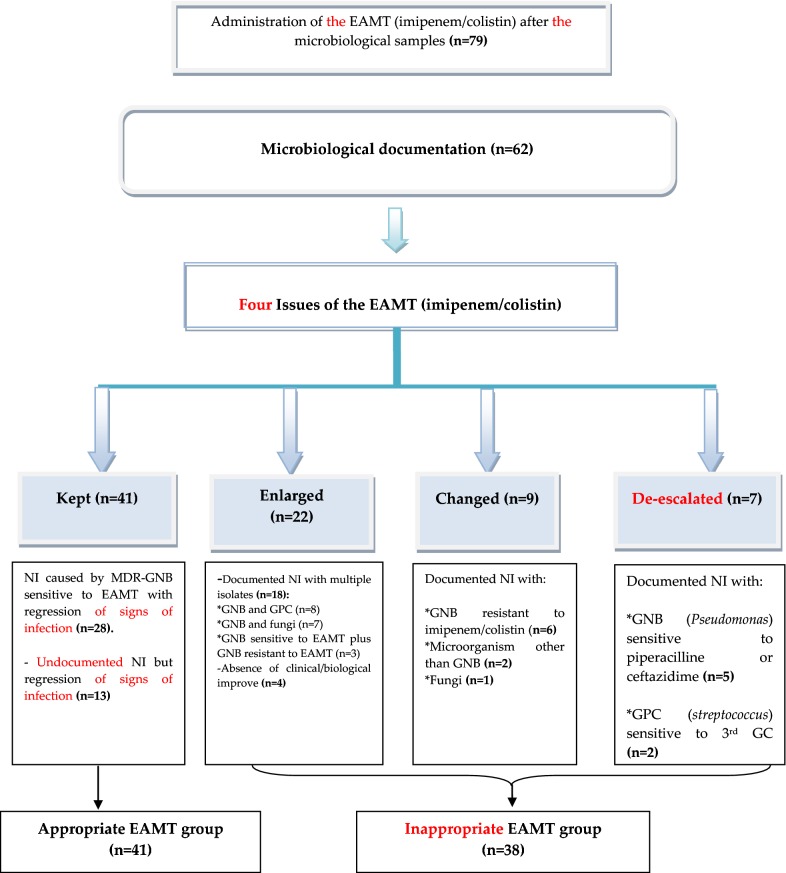
Study flowchart. NI, nosocomial infection; MDR, multi-drug resistant; GNB, Gram negative bacilli; EAMT, empiric antimicrobial therapy; GPC, gram positive cocci; 3rd GC, third generation cephalosporin

79 suspicions of NI caused by MDR-GNB were recorded in 70 patients and received imipenem/colistin as EAMT. NIs were documented in 62 cases. Note that one case may correspond to one or more NI and may be caused by one or more pathogens. Based on clinical, biological and bacteriological outcomes, EAMT with imipenem/colistin (n = 79) had four issues: kept as initially prescribed (n = 41), enlarged by addition of other antimicrobial or antifungal (n = 22), modified by other antimicrobials (n = 9) and de-escalated (n = 7). Figure 1 details the study flowchart and all clinical characteristics are summarized in Table 1.

**Table 1:** Baseline patient’s characteristics

	Studied cases (n = 79)
Age (years), mean ± SD	53 ± 17
Sex-ratio	1.15
SAPS II, mean ± SD	37 ± 15
SOFA, mean ± SD	5 ± 3
Origin, n (%)	
Emergency department	46 (58%)
Intra-hospital medical department	21 (27%)
Intra-hospital surgical department	8 (10%)
Private healthcare	4 (5%)
Reason of admission, n (%)	
Acute respiratory failure	44 (56%)
Coma	22 (27%)
Shock	6 (8%)
Infectious disease	6 (8%)
Metabolic disorder	1 (1%)
Co-morbidities, n (%)	
Diabetes	31 (39%)
Hypertension	27 (34%)
Chronic respiratory failure	27 (34%)
Cardiovascular disease	14 (18%)
Chronic renal failure	6 (7.5%)
Immunosuppression and neoplasia	6 (7.5%)
Length of stay before inclusion, days (median [IQR])	6 [4–13]
Prior antimicrobials, n (%)	64 (81%)
Steroids, n (%)	10 (13%)
Mechanical Ventilation, n (%)	77 (97.5%)
Recent surgery, n (%)^a^	14 (18%)
Tracheostomy, n (%)	33 (42%)

SD, standard derivation; SAPS, Simplified Acute Physiology Score Evaluation; SOFA, Sequential Organ Failure Assessment

^a^ During the last 6 months

### EAMT details

Hemodynamic worsening and/or persistence of infectious signs (in patients already receiving antimicrobials other than imipenem/colistin combination) were the most common reason of EAMT administration (81%). Once NI was suspected, the delay in starting EAMT (mn) did not differ between groups (appropriate EAMT: 32 mn versus inappropriate EAMT: 38 mn, p = 0.91) or between subgroups (p = 0.3).

### Microbiological results

Bacteriological results were positives in 62/79 cases within a mean delay of 5 days. Note that diagnosed NI could have one or more locations and could be caused by one or more pathogens. VAP was the major NI location and *Acinetobacter baumannii* was the main isolate. All microbiological details are displayed on Table 2.

**Table 2:** Microbiological data in study groups

Subgroups	Appropriate EAMT group (n = 41)	Inappropriate EAMT group (n = 38)		
		Enlargement (n = 22)	Change (n = 9)	De-escalation (n = 7)
Bacteriologic documentation, n	28/41	18/22	9/9	7/7
NI location				
VAP	22	11	2	4
Bacteraemia	3	7	3	2
CRI	2	5	3	0
CRB	1	1	0	1
UI	0	1	1	0
Isolates (n)				
*Acinetobacter B*	14	6	0	0
*Klebsiella pneumonia*	6	4	0	0
*Pseudomonas* spp.	5	5	3	5
*Enterobacter*	2	4	1	0
*E. coli*	0	1	0	0
*Staphylococcus*	0	4	1	0
*Enterococcus*	0	3	1	0
*Stenotrophomonas M*	0	3	2	0
*Burkholderiacepacia*	1	0	0	0
*Streptocoque* spp.	0	0	0	2
*Candida* species	0	7	1	0
Antimicrobial adjustment	Imipenem/colistin	Imipenem/colistin/Glycopeptid (n = 8)	Other betalactam (for resistance to impenem) + tygecycline, aminoglycoside or quinolone (n = 6)	Piperacilline (n = 2)
		Imipenem/colistin/antifungal (n = 7)	Glycopeptide ± aminoside (n = 2)	Ceftazidime (n = 3)
		Imipenem/colistin/Tygecycline/aminglyosides (n = 3)	Antifungal (n = 1)	Cefotaxime (n = 2)

EAMT, empiric antimicrobial therapy; NI, nosocomial infection; VAP, ventilator acquired pneumonia; CRI, catheter related infection; CRB, catheter related bacteraemia; UI, urinary infection

### Factors influencing appropriateness of EAMT

The length of stay (LOS) before NI acquisition was similar between the two groups but differed between the subgroups (mean durations (days) were at 10 [7–13], 8 [4–11], 5 [3–10] and 5 [3–7] respectively in enlargement, change, appropriate and de-escalation subgroups with p = 0.022.

Logistic regression (NB: inappropriate EAMT was the case group and appropriate EAMT was the control group) showed that LOS pre-NI acquisition > 6 days was associated with inappropriateness of EAMT (OR = 4.44, 95% CI [1.06–20.4], p = 0.05). On the other hand, chronic respiratory failure was a factor associated with appropriateness of this EAMT (OR = 0.26, 95% CI [0.075–0.93], p = 0.038) (Table 3).

**Table 3:** Factors influencing appropriateness or not of EAMT

	Appropriate EAMT (n = 41)	Inappropriate EAMT (n = 38)	p	Logistic regression results		
				OR	95% CI	p
Age, years (mean ± SD)	50 ± 18	55 ± 16	*0.18*			
Age > 50 years, n (%)	22 (53%)	26 (68%)	*0.2*	2.4	[0.67–8.51]	0.17
Sex-ratio	26/15	21/17	0.49			
SAPS II	36 ± 14	40 ± 16	0.26			
SOFA	5.2 ± 2.9	6.5 ± 3.7	*0.076*			
SOFA > 5, n (%)	14 (34%)	20 (52%)	*0.13*	1.66	[0.54–5.06]	0.36
Origin of NI acquisition						
Medical ICU	9 (22%)	14 (37%)	*0.18*	0.61	[0.10–3.64]	0.6
ED*	22 (53%)	15 (39%)	*0.20*	1.14	[0.30–4.33]	0.84
Medical department	5 (12%)	8 (21%)	0.32			
Other	5 (12%)	1 (2.6)	–			
Co-morbidities						
Diabetes	17 (41%)	14 (37%)	0.36	0.26	[0.075–0.93]	*0.038*
Chronic respiratory failure	19 (46%)	8 (21%)	*0.032*	1.42	[0.43–4.67]	0.56
Immunosuppression/neoplasia	7 (17%)	6 (16%)	0.99			
Cardiovascular disease	17 (41%)	24 (63%)	*0.079*			
Nature of suspected NI:						
VAP	33 (80%)	26 (68%)	*0.2*	0.6	[0.19–1.94]	0.40
Bloodstream infection	6 (15%)	6 (16%)	–			
CRI	2 (5%)	5 (13%)	–			
UI	0	1 (2.6%)	–			
LOS pre-NI acquisition, days (med [IQR])	5 [3–10]	8 [6–14]	0.09			
LOS pre-NI > 6 days	10 (24%)	20 (52%)	0.012	4.44	[1.06–20.4]	*0.05*
Prior antimicrobials	32 (78%)	32 (84%)	0.57			
Pre-exposure to imipenem	10 (24%)	11 (29%)	0.8			
Pre-exposure to beta-lactams (other than imipenem)	20 (49%)	18 (47%)	0.82			
Pre-exposure to colistin	7 (17%)	8 (21%)	0.77			
Pre-exposure to glycopeptides	7 (17%)	4 (10.5%)	0.52			
Recent surgery	4 (10%)	10 (26.5%)	*0.061*	1.69	[0.37–7.66]	0.49
Shock	16 (39%)	16 (42%)	0.82			
Mechanical ventilation	40 (97.5%)	37 (97.3%)	1			
Tracheostomy	17 (41%)	16 (42%)	0.81			
Current corticosteroid	5 (12%)	5 (13%)	1			

EAMT, empiric antimicrobial therapy; SD, standard derivation; SAPS, Simplified Acute Physiology Score Evaluation; SOFA, Sequential Organ Failure Assessment; ICU, intensive care unit; ED, emergency department; NI, nosocomial infection; VAP, ventilator acquired pneumonia; CRI, catheter related infection; UI, urinary infection; LOS, length of stay; IQR, interquartile range; OR, odds ratio; CI, confidence interval

Italic values indicate p < 0.05

### Impact of EAMT on a patient’s outcome

**Fig. 2:**
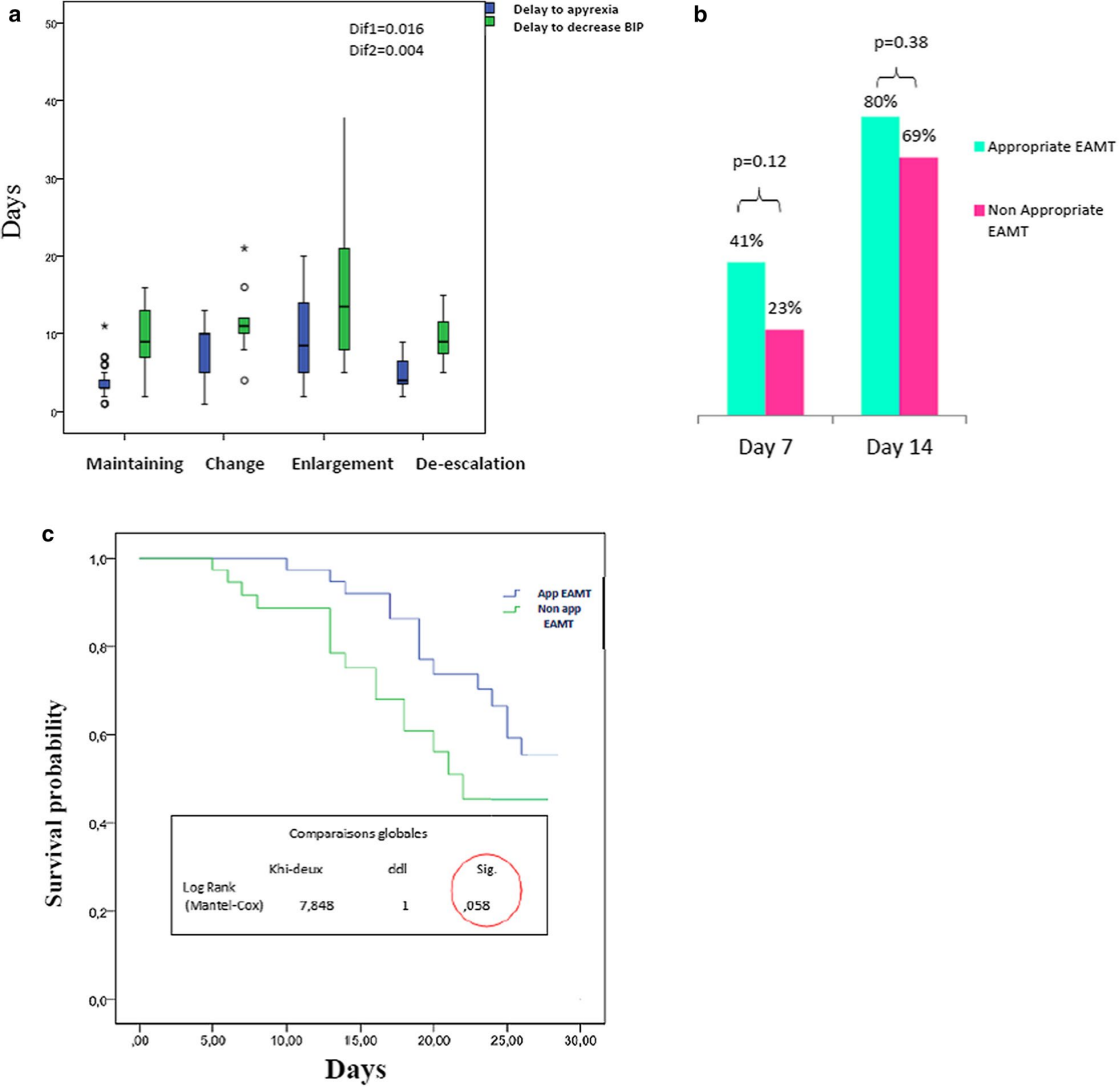
Comparison of outcome’s criteria between study groups. EAMT, empiric antimicrobial therapy; BIP, biological inflammatory parameters; dif1, significance for delay to apyrexia; dif2: significance for delay to decrease BIP
- The microbiological follow up showed that bacterial eradication rates were similar either at day 7 or at day 14 of (Fig. 2b).
- Vasopressors requirement was significantly superior in the inappropriate EAMT group with a mean duration of 8 days [6–14] versus 5 days [2–8] in the appropriate EAMT group, p = 0.026.
- There were no differences in ventilator days and in ICU stays between appropriate versus inappropriate EAMT (15 ± 5.9 vs 18 ± 7 days, p = 0.5 and 22 ± 12 vs 29 ± 15 days, p = 0.37 respectively). Between subgroups, patients with changed or enlarged EAMT had higher ventilator days and LOS.
- Thirteen patients in the appropriate EAMT group died (32%) compared to 20 (52%) in the inappropriate EAMT group with a near-significant difference (p = 0.071). Between subgroups, mortality was higher in the enlargement (59%) and modification (56%) comparatively to de-escalation (28.5%) subgroups.
- The survival analysis showed that appropriate EAMT improved survival at 30 days with a mean time of 25 days [23–28] compared to 21 days [19–24] and p = 0.058 (Fig. 2c).
- Inappropriate EAMT was significantly related to mortality (OR = 6.27, 95% CI [1.83–21], p = 0.003). When NI was acquired in ICU, the death rate doubled (OR = 2.02, 95% CI [1.37–12.12], p = 0.04) (Table 4).

- Delay to apyrexia was shorter in the appropriate EAMT group. Mean delays to apyrexia differed between subgroups; the most prolonged delay was observed with the enlargement subgroup. Likewise, the time to decrease the biological inflammatory parameters (BIP) was more rapid in the appropriate EAMT group (Fig. 2a).

**Table 4:** Factors associated with mortality by logistic regression

Varieties	Survivors (n = 46)	Died (n = 33)	OR [95% CI]	p
Inappropriate EAMT, n (%)	18 (39%)	20 (61%)	6.27 [1.83–21]	*0.003*
Age, mean ± DS^a^	52 ± 18	54 ± 16	1.15 [0.28–4.64]	0.83
SOFA^a^, mean ± DS	5.52	6.36	1.58 [0.79–2.91]	0.37
Chronic respiratory failure, n (%)	16 (35%)	11 (33%)	0.87 [0.35–2.15]	0.77
Cardiovascular disease, n (%)	19 (41%)	22 (66%)	1.43 [0.56–2.79]	0.25
Diabetes, n (%)	19 (41%)	12 (36%)	0.77 [0.27–1.96]	0.48
ICU acquired NI, n (%)	8 (17%)	15 (45%)	2.02 [1.37–12.12]	*0.044*
ED acquired NI, n (%)	20 (43%)	17 (51%)	1.16 [0.65–3.37]	0.67
Medical service acquired NI, n (%)	9 (19%)	4 (12%)	0.62 [0.01–1.84]	0.13
Shock, n (%)	11 (24%)	21 (63%)	1.96 [0.87–9.28]	0.09

EAMT, empiric antimicrobial therapy; SOFA, Sequential Organ Failure Assessment; ICU, intensive care unit; ED, emergency department; NI, nosocomial infection; OR, odds ratio; CI, confidence interval

^a^Studied variables: age > 50 years and SOFA > 5

Italic values indicate p < 0.05

## Discussion

### Main findings

EAMT was appropriate only in 52% of studied cases. A LOS before NI acquisition beyond 6 days was independently related to inappropriateness contrary to a history of chronic respiratory failure that was associated to appropriateness. Appropriate EAMT improved clinical and biological evolution and 30-day survival. Moreover, inappropriateness increased ICU risk mortality by six.

### Rational of empiric antimicrobial therapy using imipenem/colistin

Expert recommendations emphasize the quality and appropriateness of empiric antibiotics, especially in severe sepsis [17, 18]. Among the major pathogens in the cause of ICU-NI, *Pseudomonas aeruginosa* has the potential to become resistant to all antibiotics because of its ability to create bio-films and overexpression of AmpC-B-lactamases and metallo-B-lactamases MBLs [7]. *Acinetobacter* spp. has multiple mechanisms for horizontally developing and transferring resistance, the most important of which are the production of β-lactamases and the modification of aminoglycoside enzymes. For enterobacterial isolates, the spread of the strain that produces extended-spectrum β-lactamases (ESBLs) has been reported worldwide. Carbapenems have become the best and last drug for treatment; contributing to the increase of carbapenem resistance by Enterobacteriaceae (CRE). In China, carbapenem-resistant *Klebsiella pneumoniae* was the main isolate among CRE due to the production of *bla*_*IMP*−*4*_ and *armA genes* [8].

These three pathogens were the most isolates in our series, which justified the choice of imipenem/colistin combination as a first-line therapy. The screening of MDR strains can guide the clinician to initiate appropriate empiric therapy [19, 20]. Likewise, the fluid circulation of information and even the creation of a multidisciplinary communication network (bacteriologist, clinician, pharmacologist, etc.) is an approach that helps to optimize the ‘‘ best choice ‘‘ of the initial antimicrobials.

Because standard bacteriologic methods are time consuming, molecular techniques namely PCR amplifications have shown to be a rapid and reliable approach for the identification of bacterial pathogens [21–25]. Several PCR-based methods have been described to identify *P. aeruginosa* [22], *Acinetobacter baumannii* [23, 24], and ESBL-producing *Escherichia coli*  [25]. Most studies in this topic concur that this novel strategy offers a rapid (< 1.5 h) tool for clinicians to initiate an appropriate treatment earlier compared to phenotypic methods. In Tunisia, PCR amplification methods are not performed routinely; it is done during investigation of an epidemic infection [26].

The time to obtain bacteriological results was overall 5 days in our study and in the absence of the practise of these novel techniques routinely, an empiric prescription of imipenem/colistin remains judicious.

The subsequent control of EAMT is a crucial step in the best practices of antimicrobials use. Leone et al. [27] showed that de-escalation was safe and associated with lower antibiotic use and shorter antibiotic duration; and theoretically a beneficial effect on the MDR emergence [27, 28]. Leone et al. [27] reported an appropriateness rate of 89% in cases [including de-escalation (42%)] [27]. In our series, de-escalation was only possible in 7/79 (9%) of initial prescriptions, given the predominance of MDR strains. We opted for considering de-escalation as inappropriate because this “excessive” regimen threatens the future efficiency of the current available antibiotics.

Nonetheless, De Bus et al. [29] analyzed the effect of de-escalation on 478 prescriptions for anti-*Pseudomonas* antibiotics and found no lower levels of resistant strains after exposure to broad-spectrum B-lactams.

### Factors influencing appropriateness of EAMT

Several observational studies demonstrated that the use of antimicrobial combinations is superior than monotherapy [30, 31]. The probable infection site is also an influencing factor involved in the appropriateness of empiric therapy. It should guide the choice of antimicrobials capable of reaching the therapeutic concentrations in the infected tissues and fluids. An American multi-center cohort study revealed that nosocomial pneumonia and urinary tract infection had the greatest variation for appropriate use (50–100% for nosocomial pneumonia and 64.9–100% for urinary tract infection) [32]. In our series, no influence was showed in the probable site of NI.

Chronic co morbidities, immune status and social history (frequent travel, incarceration, illicit drug use…) should also be taken into account [33]. We found that a history of chronic respiratory failure (CRF) was significantly associated with appropriate EAMT. Even if it is considered as an unexpected result; patients with CRF could be carried by MDR (not necessarily identifiable) due to their frequent hospitalizations for exacerbation. In this case, imipenem/colistin the combination would play a key role in improvement.

Otherwise, hospitalization > 6 days was an independent factor associated with inappropriateness in our series. This should encourage the reconsideration of non-GNB or GNB with a special susceptibility profile in the choice of EAMT. That concerned mainly GPC or strains with potential acquisition of resistance to imipenem (such *Pseudomonas, acinetobacter*…) or selected organisms with a natural resistance to colistin (*Proteus, Providencia.*..). No effect was found for previous antimicrobial use, recent surgery, shock or age. For Paul et al. [31] shock was associated with adequate empiric therapy. Willemsen et al. [34] showed that the use of quinolones was the only determinant of inappropriateness [34].

Several studies have emphasized the ‘‘timing’’ for antimicrobial initiation [35, 36]. For Luna, et al. [29], patients classified in the inappropriate arm received empiric therapy on a mean delay of 28.6 ± 5.8 h versus 12.5 ± 4.2 h, p < 0.01 in inappropriate arm. Leibman et al. developed a prediction score in order to optimize the time and the decision to initiate appropriate therapy for CRE including six items [37]. A score of ≥ 32 predicted “high CRE risk” and thus indicated an empiric therapy with 90% of negative predictive value [37].

The time to initiate empiric therapy in our series was rapid and similar for both groups and therefore it wasn’t studied as an influential factor.

The retrospective study (2005–2010) of Al-Dorzi et al. [38] assessing the impact of empiric antimicrobial in *Acinetobacter* bacteraemia identified that female sex, admission during 2008–2010 compared to 2005–2007, mechanical ventilation and age were associated with appropriate therapy [38].

### Impact on patient’s outcome

The poor impact of inappropriate initial antimicrobial was widely demonstrated [31, 39–46]. A Spanish multi-center study found that the mortality attributable to appropriate empiric therapy was 16.2% versus 24.7% in patients who received inappropriate therapy [41]. A meta-analysis (70 studies) evaluated the efficacy of appropriate empiric antimicrobial therapy for sepsis, showed that inappropriateness of initial therapy was associated with significant mortality (OR = 2.05, 95% CI [1.69–2.49]) [31]. The large prospective study of Paul et al. [42] including 2000 medical/surgical patients showed that the hospital mortality rate in infected patients who received initial inappropriate therapy was statistically higher 52.1% versus 12.2% with RR = 4.26, 95% CI [3.52–5.15]; p < 0.001.

Another large systematic review (57 studies) [46] showed that appropriate empiric therapy was associated with a lower mortality risk (OR adjusted = 0.43, 95% CI 0.23–0.83) and lower therapeutic failure (OR = 0.22, 95% CI 0.14–0.35). In contrast, inappropriate empiric therapy increased mortality risk (OR = 3.30, 95% CI 2.42–4.49) [46]. A systematic review and meta-analysis of mortality in patients infected with carbapenem-resistant *Klebsiella pneumoniae* showed the pooled mortality was 42.14% versus 21.16% in those infected with carbapenem-susceptible *K. pneumonia* [47]. This difference was due to an inappropriate empiric therapy with very limited therapeutic options [47].

In our series, despite the inclusion of a de-escalation subgroup (with the lowest mortality) in the inappropriate EAMT arm, inadequate empiric therapy was significantly related to mortality (OR = 6.27, 95% CI [1.83–21], p = 0.003). In addition, the acquisition of NI in ICU was also a significant factor related to mortality. This is explained by the fact that the strains selected in ICU are the most concerned by the multi-resistance to antimicrobials, the high risk of invasive candidiasis, a greater severity and a heavier underlying co-morbidity. All these factors are likely to worsen the patient’s prognosis.

Nevertheless, Zaragoza et al. [48] and Falagas et al. [43] did not find a significant relationship between inappropriate empiric therapy and mortality.

Furthermore, inappropriate empiric antimicrobials were associated with higher hospital costs ($ 51,977, [$ 34,644–$ 69,311]) and a prolonged stay hospital (21, [13–21] days) in comparison with an appropriate empiric therapy ($ 40,187, [$ 25,982–$ 54,392]) and (18, [9–24] days) [46].

### Strength and weakness

it is estimated that the strong point of this study is its originality; the imipenem/colistin combination is the antimicrobial therapy used as first choice in front of a nosocomial septic, but is it really the best choice? On the other hand, the therapeutic arsenal with proven efficacy against MDR pathogens is very limited and therefore these molecules should to be preserved by rational use. Hence the need for the assessment of its clinical impact to which this study has responded. To the best of our knowledge, this is the first to evaluate the combination imipenem/colistin as empiric therapy.

The weak points are the mono-centric design and the small sample studied. Another point could be the source of an evaluation bias; which is the inclusion of the de-escalation subgroup in the inappropriate EAMT. This is explained by the fact that in this subgroup, EAMT was effective against isolated organisms. However, because of the broad spectrum of this association, a source of selection for resistant mutants, its prescription was considered unjustified. That was the reason why we chose to integrate this subgroup into the inappropriate arm.

## Conclusions

Despite the high proportion of MDR-GNB in the cause of nosocomial sepsis, empiric antimicrobial therapy using imipenem-colistin was only appropriate in half of cases and increased mortality. It would therefore be judicious to revise this empiric therapy with the elaboration of therapeutic regimens according to the duration before NI acquisition, its location and colonization with MDR bacteria. A multi-disciplinary approach involving clinicians, microbiologist and pharmacologist is paramount to ensure a rational prescription of antimicrobials. The introduction of rapid diagnostic tests, such as PCR techniques, would be a necessary step to reach rapidly and surely the appropriateness of empiric therapy.

## Abbreviations

EAMT: empiric antimicrobial therapy
NI: nosocomial infection
ICU: intensive care unit
MDR: multidrug-resistant
GNB: Gram-negative bacilli
ESBL: extended-spectrum beta lactamase
LOS: length of stay
BIP: biological inflammatory parameters
GPC: Gram positive cocci
3rd GC: third generation cephalosporin
SAPS: Simplified Acute Physiology Score Evaluation
SOFA: Sequential Organ Failure Assessment
ED: emergency department,
AP: ventilator acquired pneumonia
CRI: catheter related infection
UI: urinary infection
SD: standard derivation
IQR: inter quartile range
OR: odds ratio
CI: confidence interval
